# Genetic risk for schizophrenia is associated with increased proportion of indirect connections in brain networks revealed by a semi-metric analysis: evidence from population sample stratified for polygenic risk

**DOI:** 10.1093/cercor/bhac256

**Published:** 2022-07-14

**Authors:** S I Dimitriadis, G Perry, T M Lancaster, K E Tansey, K D Singh, P Holmans, A Pocklington, G Davey Smith, S Zammit, J Hall, M C O’Donovan, M J Owen, D K Jones, D E Linden

**Affiliations:** Neuroscience and Mental Health Research Institute (NMHI), College of Biomedical and Life Sciences, Cardiff University, Maindy Road CF24 4HQ, Cardiff, Wales, UK; Cardiff University Brain Research Imaging Centre (CUBRIC), School of Psychology, College of Biomedical and Life Sciences, Cardiff University, Maindy Road CF24 4HQ, Cardiff, Wales, UK; Division of Psychological Medicine and Clinical Neurosciences, MRC Centre for Neuropsychiatric Genetics and Genomics, Cardiff School of Medicine, Cardiff University, Maindy Road CF24 4HQ, Cardiff, Wales, UK; Neuroinformatics Group, School of Psychology, College of Biomedical and Life Sciences, Cardiff University, Maindy Road CF24 4HQ, Cardiff, Wales, UK; Cardiff University Brain Research Imaging Centre (CUBRIC), School of Psychology, College of Biomedical and Life Sciences, Cardiff University, Maindy Road CF24 4HQ, Cardiff, Wales, UK; Neuroscience and Mental Health Research Institute (NMHI), College of Biomedical and Life Sciences, Cardiff University, Maindy Road CF24 4HQ, Cardiff, Wales, UK; Cardiff University Brain Research Imaging Centre (CUBRIC), School of Psychology, College of Biomedical and Life Sciences, Cardiff University, Maindy Road CF24 4HQ, Cardiff, Wales, UK; Department of Psychology, Bath University, Claverton Down BA2 7AY, Bath, Wales, UK; MRC Integrative Epidemiology Unit (IEU), University of Bristol, Queens Road BS8 1QU, Bristol, Wales, UK; Cardiff University Brain Research Imaging Centre (CUBRIC), School of Psychology, College of Biomedical and Life Sciences, Cardiff University, Maindy Road CF24 4HQ, Cardiff, Wales, UK; Division of Psychological Medicine and Clinical Neurosciences, MRC Centre for Neuropsychiatric Genetics and Genomics, Cardiff School of Medicine, Cardiff University, Maindy Road CF24 4HQ, Cardiff, Wales, UK; Division of Psychological Medicine and Clinical Neurosciences, MRC Centre for Neuropsychiatric Genetics and Genomics, Cardiff School of Medicine, Cardiff University, Maindy Road CF24 4HQ, Cardiff, Wales, UK; MRC Integrative Epidemiology Unit (IEU), University of Bristol, Queens Road BS8 1QU, Bristol, Wales, UK; Population Health Sciences, Bristol Medical School, University of Bristol, 1-5 Whiteladies Road BS8 1NU, Bristol, Wales, UK; Division of Psychological Medicine and Clinical Neurosciences, MRC Centre for Neuropsychiatric Genetics and Genomics, Cardiff School of Medicine, Cardiff University, Maindy Road CF24 4HQ, Cardiff, Wales, UK; Population Health Sciences, Bristol Medical School, University of Bristol, 1-5 Whiteladies Road BS8 1NU, Bristol, Wales, UK; Neuroscience and Mental Health Research Institute (NMHI), College of Biomedical and Life Sciences, Cardiff University, Maindy Road CF24 4HQ, Cardiff, Wales, UK; Division of Psychological Medicine and Clinical Neurosciences, MRC Centre for Neuropsychiatric Genetics and Genomics, Cardiff School of Medicine, Cardiff University, Maindy Road CF24 4HQ, Cardiff, Wales, UK; Neuroscience and Mental Health Research Institute (NMHI), College of Biomedical and Life Sciences, Cardiff University, Maindy Road CF24 4HQ, Cardiff, Wales, UK; Division of Psychological Medicine and Clinical Neurosciences, MRC Centre for Neuropsychiatric Genetics and Genomics, Cardiff School of Medicine, Cardiff University, Maindy Road CF24 4HQ, Cardiff, Wales, UK; Neuroscience and Mental Health Research Institute (NMHI), College of Biomedical and Life Sciences, Cardiff University, Maindy Road CF24 4HQ, Cardiff, Wales, UK; Division of Psychological Medicine and Clinical Neurosciences, MRC Centre for Neuropsychiatric Genetics and Genomics, Cardiff School of Medicine, Cardiff University, Maindy Road CF24 4HQ, Cardiff, Wales, UK; Cardiff University Brain Research Imaging Centre (CUBRIC), School of Psychology, College of Biomedical and Life Sciences, Cardiff University, Maindy Road CF24 4HQ, Cardiff, Wales, UK; Neuroscience and Mental Health Research Institute (NMHI), College of Biomedical and Life Sciences, Cardiff University, Maindy Road CF24 4HQ, Cardiff, Wales, UK; Cardiff University Brain Research Imaging Centre (CUBRIC), School of Psychology, College of Biomedical and Life Sciences, Cardiff University, Maindy Road CF24 4HQ, Cardiff, Wales, UK; Division of Psychological Medicine and Clinical Neurosciences, MRC Centre for Neuropsychiatric Genetics and Genomics, Cardiff School of Medicine, Cardiff University, Maindy Road CF24 4HQ, Cardiff, Wales, UK; Population Health Sciences, Bristol Medical School, University of Bristol, 1-5 Whiteladies Road BS8 1NU, Bristol, Wales, UK; School for Mental Health and Neuroscience, Faculty of Health, Medicine and Life Sciences, Maastricht University, Universiteitssingel 40 UNS40 6229 ER, Maastricht, The Netherlands

**Keywords:** diffusion magnetic resonance imaging (dMRI), tractography, schizophrenia, structural brain networks, genetic risk for schizophrenia, semi-metric percentage, brain connectomics, Avon Longitudinal Study of Parents and Children (ALSPAC)

## Abstract

Research studies based on tractography have revealed a prominent reduction of asymmetry in some key white-matter tracts in schizophrenia (SCZ). However, we know little about the influence of common genetic risk factors for SCZ on the efficiency of routing on structural brain networks (SBNs). Here, we use a novel recall-by-genotype approach, where we sample young adults from a population-based cohort (ALSPAC:*N* genotyped = 8,365) based on their burden of common SCZ risk alleles as defined by polygenic risk score (PRS). We compared 181 individuals at extremes of low (*N* = 91) or high (*N* = 90) SCZ-PRS under a robust diffusion MRI-based graph theoretical SBN framework. We applied a semi-metric analysis revealing higher SMR values for the high SCZ-PRS group compared with the low SCZ-PRS group in the left hemisphere. Furthermore, a hemispheric asymmetry index showed a higher leftward preponderance of indirect connections for the high SCZ-PRS group compared with the low SCZ-PRS group (*P*_FDR_ < 0.05). These findings might indicate less efficient structural connectivity in the higher genetic risk group. This is the first study in a population-based sample that reveals differences in the efficiency of SBNs associated with common genetic risk variants for SCZ.

## Introduction

Schizophrenia (SCZ) is a highly heritable disorder involving a combination of rare and common risk alleles distributed across the genome (Sullivan et al. 2012). To date, the vast majority of risk alleles identified are common; although individual alleles confer very small effect sizes on risk (typically odds ratios <1.1), cumulatively common alleles account for at least a third of genetic liability (Schizophrenia Working Group of the Psychiatric Genomics Consortium 2014). The total burden of common risk alleles for SCZ carried by an individual can be estimated by a metric known as a polygenic risk score (PRS) (International Schizophrenia Consortium et al. 2009). PRS are created by summing for an individual the number of risk alleles identified from genome-wide association studies (0, 1, or 2), weighted by their individual effect size (for further details see Lancaster et al.  2016; Choi et al. 2020). Previous studies have combined SCZ-PRS profiles with neuroimaging measures in healthy individuals to detect alterations in brain structure and function that potentially are key mechanisms of SCZ pathogenesis (Miller et al. 2018; Lancaster et al. 2019). These studies have concluded that subcortical structural abnormalities observed in SCZ show little or no genetic overlap with SCZ common variant liability (Ranlund et al. 2018; Lancaster et al. 2019; Lv et al. 2021; Lancaster et al. 2022).

Recent genetic imaging studies shed light on how risk alleles for SCZ can affect brain and behavior in people stratified for either PRS (Lancaster et al. 2019) or genomic copy number variants (CNVs; Stefansson et al. 2014). When applied in population cohorts, both approaches can elucidate alterations in brain development both structurally and functionally that cannot be secondary to disease effects. A diffusion-MRI (dMRI) study of adult participants carrying neuropsychiatric risk CNVs investigated the role of shape and microstructural organization white matter (WM) structures in the brain development. They found that midline WM structures involved to this abnormal brain development pattern associated with a genetic risk for SCZ and learning disability (Drakesmith et al. 2019). A large-scale study including both cases and healthy control based on UK Biobank found a strong association between PRS with microstructural MRI metrics at both global and local level of cortex including the thalamus, basal ganglia, and hippocampus, and extensively in WM tracts (Stauffer et al. 2021). Another study, focusing on the ability of SZ PRS's to predict a first episode of psychosis, found no association between SCZ-PRS and fractional anisotropy (FA) and mean diffusivity (MD) (Simões et al. 2020).

Brain asymmetries are widespread in vertebrates and invertebrates and can arise via a number of genetic, epigenetic, and neural mechanisms (Corballis 2014). In humans, structural and functional brain asymmetries are the characteristic of the healthy brain (Rentería 2012) and are associated with functions such as language (dominance of left hemisphere, Corballis 2014) and spatial attention (dominance of right hemisphere, Iturria-Medina et al. 2011; Cai et al. 2013). In contrast, attenuation of this brain asymmetry has been reported in SCZ (Oertel-Knöchel and Linden 2011), both in terms of reduced structural (Baker et al. 2014; Zhang et al. 2015) and functional asymmetry, compared with controls, with intermediate values in unaffected relatives (Oertel et al. 2010). A recent study using UK Biobank MRI data revealed associations of regional brain asymmetries linked to executive functions and language with polygenic risks for both ASD and schizophrenia (Sha, Schijven, Carrion-Castillo, et al. 2021a).

Altered brain asymmetry has also been linked to symptoms of SCZ, which may reflect the attenuation of left-hemisphere dominance for language processing (Crow 2008). Both structural and functional imaging studies have suggested that SCZ is associated with reduced left-ward lateralization of language functions (Kircher et al. 2002; Sumich et al. 2002) and that this attenuation of left-hemispheric dominance is associated with the progression of the disease (Lam et al. 2012).

Complementary findings to the above studies have also been reported for measures of inter- and intrahemispheric connectivity. Diffusion tensor imaging (DTI) in SCZ revealed reduced connectivity across different progressive stages of the disorder (Wheeler and Voineskos 2014), in first-episode subjects (Zhang et al. 2015), in high-risk subjects (Carletti et al. 2012), and in people with high schizotypy scores (Smallman et al. 2014). These aberrant structural connectivity patterns are thus possible neurobiological biomarkers across the SCZ spectrum (Ribolsi et al. 2009). In particular, FA patterns in the uncinate fascicle showed rightward asymmetry in SCZ patients, but not in healthy controls (Miyata et al. 2012). Another DTI study examined FA patterns of the superior occipito-frontal fascicle in SCZ patients and healthy controls. They reported an absence of normal leftward brain asymmetry in patients (Kunimatsu et al. 2008). Finally, a DTI study dedicated to anterior and posterior cingulum revealed an attenuation of leftward asymmetry of FA in the anterior cingulum bundle in SCZ patients compared with healthy controls (Fujiwara et al. 2007).

Recent DTI studies also reported diffusion abnormalities in the corpus callosum of SCZ patients, which may reflect changes that influence axonal transmission velocities, emphasizing neural timing abnormalities as a potential etiology for SCZ (Whitford et al. 2010; Del Re et al. 2019). A DTI study with SCZ patients and their relatives reported reduced interhemispheric connectivity in both groups compared with controls, which predicted interhemispheric transfer time (Whitford et al. 2010) and psychotic symptoms.

This accumulating evidence for altered hemispheric connectivity and asymmetry in schizophrenia motivated us to perform an integrated investigation of the effects of genetic risk (Schijven et al. 2022). We used semi-metric analysis, a parsimonious way of evaluating the efficiency of neural pathways (Peeters et al. 2015; Simas et al. 2015). The semi-metric analysis is based on the estimation of shortest-path lengths, delineating direct paths of one step between ROIs (when a connection exists between a pair of ROIs) compared with indirect paths where 2 nodes are joined through one or more intermediary ROIs (when no connection exists between a pair of ROIs). Semi-metric analysis has been applied to functional connectivity data and has revealed alterations in different neurodevelopmental disorders (Peeters et al. 2015; Simas et al. 2015; Suckling et al. 2015; Guo et al. 2019).

We present the first DTI study applying semi-metric analysis of structural connectivity, based on diffusion MRI data in a SCZ genetic risk group. Higher semi-metric scores indicate connectivity through a higher proportion of indirect paths, which can be interpreted as a higher level of redundant interactions and dispersed communication between brain areas (Simas et al. 2015). Redundancy is an important attribute in information flow between large-scale brain networks, but it comes at the cost of reduced efficiency (Avena-Koenigsberger et al. 2017). A semi-metric edge occurs when the shortest topological path between 2 regions is a circuitous path involving additional regions rather than the direct path between them, which, in the terminology of graph theory, can also be described as a reduction of transitivity (Simas and Rocha 2012; Chong et al. 2020).

This semi-metric behavior supports a high degree of redundancy and between-network interactions in the brain (Simas et al. 2015; Simas and Suckling 2016). Network integration via indirect routing increases the dispersion of information flow, decreases the efficiency of information flow, and possibly also the risk for atypical information processing (Guo et al. 2019).

We hypothesize that the efficiency of intra- and interhemispheric connectivity, measured by the proportion of indirect pathways revealed by semi-metric analysis, will be reduced in individuals with high polygenic risk for SCZ. Thus, we expect higher semi-metric values for the high SCZ-PRS group compared with the low SCZ-PRS group.

## Material and methods

All analyses were performed using MATLAB (MATLAB and Statistics Toolbox Release, version 2019b; The MathWorks, Inc., MA, Unites States), unless otherwise stated.

### PRS stratification

Participants were recruited by the ALSPAC (Avon Longitudinal Study of Parents and Children) birth cohort by polygenic risk for Schizophrenia. This broader cohort consisted of 14,062 children born to women residing in the Avon Health Authority area with the period starting from 1991 April 1 to 1992 December 31 (http://www.bristol.ac.uk/alspac/; available at http://www.bristol.ac.uk/alspac/researchers/access/). Pregnant women resident in Avon, the United Kingdom, with expected dates of delivery from 1991 April 1 to 1992 December 31 were invited to take part in the study. The initial number of pregnancies enrolled is 14,541 (for these at least one questionnaire has been returned or a “Children in Focus” clinic had been attended by 19/07/99). Of these initial pregnancies, there was a total of 14,676 fetuses, resulting in 14,062 live births and 13,988 children who were alive at 1 year of age (Boyd et al. 2013; Fraser et al. 2013; Northstone et al. 2019). PRSs for Schizophrenia (SCZ-PRS) have been estimated for *n* = 8169 children following a normal distribution.

SCZ-PRS were constructed following the methods described in detail by the International Schizophrenia Consortium (International Schizophrenia Consortium et al. 2009) using results from the Psychiatric Genomics Consortium (PGC) SCZ genome-wide association studies (GWAS) (Schizophrenia Working Group of the Psychiatric Genomics Consortium 2014). For further details, see our study (Lancaster et al. 2019).

For a recent multi-modal neuroimaging study, we attempted to recruit 200 subjects from the extremes of this distribution in order to create two groups of 100 subjects with high and low SCZ-PRS groups, matched on sex (Lancaster et al. 2019). The study was approved by the Central Bristol Research Ethics Committee (13/SW/0170) and the local research ethics committees (listed at http://www.bristol.ac.uk/alspac/researchers/research-ethics/).

All participants were recruited from ALSPAC on the basis of their PRS for Schizophrenia. The ALSPAC team sent out 1241 invitations in total (470 to the low and 771 to the high SCZ-PRS group). Individuals were excluded if they were receiving any psychotropic medication. We ultimately assessed 203 individuals—99 (52 female, 47 male) individuals with low SCZ-PRS and 104 individuals (52 female, 52 male) with high SCZ-PRS age-matched from either tail of the SCZ-PRS distribution from a large genotyped population (see Fig. 1 in Lancaster et al. 2019). The mean group z-PRS was above 1.5 (1.42 higher than the mean PRS for the high, 1.71 lower than the mean PRS for the low group). The two groups did not differ on age (low SCZ-PRS 22 years and 1 month ±10 months, high SCZ-PRS 22 years and 2 months ±8 months with a *P*-value = 0.33, Wilcoxon rank-sum test). Psychotic experiences (hallucinations, delusions, or experiences of thought interference) were assessed using the semi-structured Psychosis-Like Symptom Interview at 18 years of age. For further details, see Table 1 in Lancaster et al. (2019). Ethical approval for the study was obtained from the ALSPAC Ethics and Law Committee and the Local Research Ethics Committees (NHS Haydock REC: 10/H1010/70). Informed consent for the use of data collected via questionnaires and clinics was obtained from participants following the recommendations of the ALSPAC Ethics and Law Committee. For further details about this cohort and the multimodal imaging protocol, see (Lancaster et al. 2019; Sharp et al. 2020). Study data were collected and managed using REDCap (Research Electronic Data Capture) electronic data capture tools hosted at CUBRIC Neuroimaging Centre (Harris et al. 2009, 2019). REDCap is a secure, web-based software platform designed to support data capture for research studies, providing (i) an intuitive interface for validated data capture; (ii) audit trails for tracking data manipulation and export procedures; (iii) automated export procedures for seamless data downloads to common statistical packages; and (iv) procedures for data integration and interoperability with external sources.

**Fig. 1:**
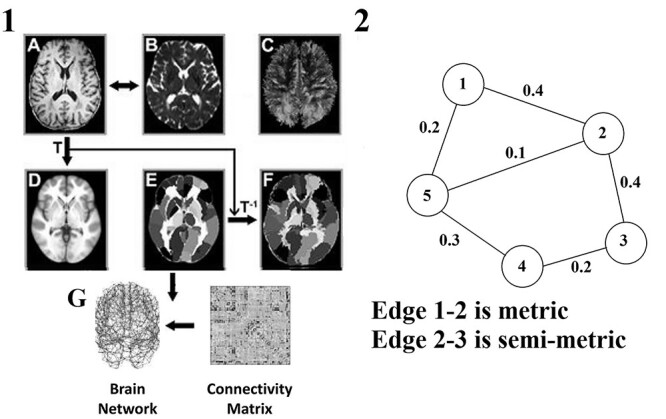
A flowchart of brain WM network construction using diffusion magnetic resonance imaging (MRI). 1) The *T*_1_-weighted image A) was aligned to the b0 image B) through a linear registration. 2) The co-registered *T*_1_-weighted image in the native DTI space was mapped into the AAL *T*_1_ template in the MNI space D) using a nonlinear transformation matrix T. 3) The inverse transformation of *T*_1_ (T^−1^) was applied to the contracted AAL atlas in the MNI space E), resulting in individual-specific parcellation in the DTI native space. (4) The reconstruction of the whole-brain WM fibers (C) was performed using deterministic tractography. (5) The weighted networks of each subject G) were created by computational steps described in (Guo et al. 2019) and Section 2.3.2. 2) An example of semi-metric analysis in a simple network (for further explanation see Section 2.3.3). Characterization of edge 1–2: Nodes 1 and 2 are connected via the indirect path 1 → 5 → 2. The sum of weights along this indirect path is 0.3 (0.2 + 0.1) which is smaller than the direct path between node 1 and 2 (0.4). Therefore, the edge 1–2 is called a metric. Characterization of edge 2–3: Nodes 2 and 3 are connected via the direct path 2 → 3. The weight of this direct path is 0.4 which is lower than the indirect path 2 → 5 → 4 → 3 with a total weight equaling 0.6 (0.1 + 0.3 + 0.2). Therefore, the edge 2–3 is called semi-metric.

Please note that the study website contains details of all the data that are available through a fully searchable data dictionary and variable search tool and reference the following webpage: http://www.bristol.ac.uk/alspac/researchers/our-data/.

### Psychotic experiences and cognition

We assessed psychotic experiences (hallucinations, delusions, or experiences of thought interference) by adopting the semi-structured Psychosis-Like Symptom Interview at 18 years of age (Zammit et al. 2013). Individuals were considered to have a psychotic experience if rated as having one or more suspected and/or definite psychotic experiences at 18 years of age (pliks18). Individuals were administered the short form Wechsler Intelligence Scale for Children (WISC-III) at 8 years of age (Wechsle et al. 1992). Scores for verbal, performance, and total IQ were entered in a regression analysis with brain asymmetry index (BAI; see Section 2.5 for further details).

### Data

All MRI data were acquired in the Cardiff University Brain Research Imaging Centre (CUBRIC) on a 3T GE Signa HDx system (General Electric, Milwaukee, USA).

#### Structural MRI scanning

Participants had already had MRI scanning before the commencement of the recall by genotype. *T*_1_-weighted structural scans were acquired using an oblique axial, 3D fast-spoiled gradient recalled sequence (FSPGR) with the following parameters: TR = 7.9 ms, TE = 3.0 ms, inversion time = 450 ms, flip angle = 20°, and 1-mm isotropic resolution, with a total acquisition time of ~7 min.

**Table 1:** Metrics used in connectivity matrices.

Metric	Abbreviation
Fractional anisotropy	FA
Mean diffusivity	MD
Radial diffusivity	RD
Number of streamlines	NS
Percentage of streamlines	PS
Streamline density	SLD
Tract volume	TV
Tract length	TL
Euclidean distance between nodes	ED

#### Diffusion MRI scanning

High-angular resolution diffusion-weighted imaging (HARDI) data were acquired in the Cardiff University Brain Research Imaging Centre (CUBRIC) on a 3T GE Signa HDx system (General Electric, Milwaukee, USA) using a cardiac-gated, peripherally gated twice-refocused spin-echo Echo Planar Imaging (EPI) sequence, with effective TR/TE of 15R-R intervals/87 ms. Sets of 60 contiguous 2.4-mm-thick axial slices were obtained, with diffusion-sensitizing gradients applied along 30 isotropically distributed (Jones et al. 1999) gradient directions (*b* = 1200 s/mm^2^). For further details of the MRI protocol, see (Bracht et al. 2016). We performed the analyses described below separately for the DWIs collected with different diffusion weighting techniques.

#### Tractography

We performed whole-brain tractography using ExploreDTI-4.8.6 (Leemans and Jones 2009). Constrained Spherical Deconvolution (CSD; Tournier et al. 2004) was used to estimate the fiber orientation distribution function. In the tractography algorithm, the seed point resolution was (2 × 2 × 2 mm^3^), the step size was 1 mm, the angle threshold was 30°, and the fiber length range was 50–500 mm.

### Graph generation

We constructed graphs using 9 different diffusion Magnetic Resonance Imaging (dMRI)-based metrics derived from ExploreDTI (Leemans and Jones 2009). The complete list of these metrics tabulated in Table 1 are: FA, MD, radial diffusivity, number of streamlines, streamline density, percentage of streamlines, tract volume, tract length and Euclidean distance between the nodes. We normalized all graphs so that the maximum edge weight in each graph was equal to 1. We also set the elements in the main diagonal of the connectivity matrices equal to 0, since they are related to self-connections of every brain area.

#### Node definition

We employed the standard Automated Anatomical Labeling (AAL) atlas (Tzourio-Mazoyer et al. 2002) to define the 90 cortical and subcortical areas of the cerebrum (45 areas per hemisphere) that correspond to the nodes of the structural brain networks (SBNs). The WM tracts interconnect those brain areas and correspond to the connections, or edges, of the produced SBNs. The network generation was performed in ExploreDTI-4.8.6 (Leemans and Jones 2009). This process resulted in a total of 9 SBNs of size 90 × 90 employing the diffusion weighting images for each participant. Every SBN had edges weighted by a different metric derived by averaging along the corresponding WM tract that interconnect every pair of brain areas.

Figure 1.1 illustrates the flowchart of the construction of the aforementioned diffusion MRI-based SBNs.

#### Integrated metric-based SBNs

**Fig. 2:**
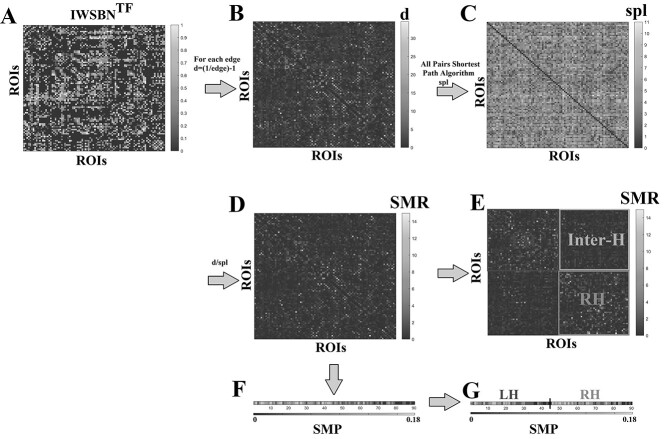
Outline of the semi-metric analysis at various spatial scales. A) Original integrated weighted SBN topological filtered with our approach (IWSBN). B) The distance matrix d equals for every edge the weight from the IWSBN^TF^ as *d_ij_* = (1/edge) − 1. C) The all pairs shortest path matrix. D) The estimated SMR matrix of size 90x90 (ROIs × ROIs) from which the global scale of SMR index and the fine edge-scale are estimated. SMR is estimated as the ratio of every cell between the matrices as it is defined in equation (2). E) We reordered the SMR matrix to underline the estimated SMR over subgraphs defined by ROIs located within the same hemisphere (left or right) and over the subgraph that encapsulates interhemispheric links (Inter-H). SMR index is estimated for the left hemisphere, right hemisphere, interhemispheric links, and for the ratio of left/right hemisphere introducing a BAI. Each hemispheric brain network is a subgraph of size 45 × 45 and was obtained eliminating interhemispheric connections. The interhemispheric subnetwork (top right corner) also has a dimension of 45 × 45. F) The estimated SMP index is derived from the integration of information from SMR matrix into ROI/node level. SMP index is a vector of size equals to 1 × 90. The global scale of SMP index and the fine node-scale are estimated. G) We reordered the SMR vector such as to distinct brain areas over the left or right hemisphere. SMP index is estimated for the left and right hemispheres.

Recently, we proposed an integrated approach of the 9 aforementioned SBNs into a single graph that combines the information tabulated within every single SBN. The algorithm is described in detail within our previous studies (Dimitriadis, Antonakakis, et al. 2017a; Dimitriadis, Drakesmith, et al. 2017b; Dimitriadis, Salis, et al. 2017c; Messaritaki et al. 2019), where using repeat scans sessions in a cohort using the same protocol and scanner as in our ALSPAC study; we showed that the integrated SBNs led to more reproducible network metrics at both global and local level than the single-metric dependent SBNs. However, this method was developed after the formulation of the analysis plan that we originally submitted to ALSPAC, and its application here is therefore to be regarded as a secondary exploratory analysis. Following our analytic pipeline (Dimitriadis, Drakesmith, et al. 2017b), for each participant, we describe for clarity in brief the basic steps of this analysis:

(1) we first applied data-driven topological filtering, using the Orthogonal Minimal Spanning Tree (OMST) algorithm (Dimitriadis, Antonakakis, et al. 2017a; Dimitriadis, Drakesmith, et al. 2017b; Dimitriadis, Salis, et al. 2017c), to every single SBN to select the edges that maximize the difference of the formula (Global Efficiency—Cost) while maintaining the connectedness of the SBN. The benefit of the method lies in its data-driven approach. Furthermore, there is no need to impose an arbitrary threshold to the graph edges, which ensures that both strong and weak edges are treated equally, which is not the case for arbitrary absolute or proportional thresholding schemes.
(2) we then adopted the graph diffusion-distance metric (Hammond et al. 2013) to quantify the distance between every pair of the 9 individual SBNs to maximize the information provided by each metric. This metric gives us the complementarity of the 9 SBNs via an information theoretic approach. The metric that is more informative across the nine gets a higher weight compared to the rest and the opposite is true for metrics that are highly correlated with the rest of the eight metrics. Finally, the weights are normalized to get a summation equal to 1, and they are linearly combined to form the so-called *integrated weighted SBN topologically filtered* (*IWSBN^TF^*; see Fig. 2A), one per participant (Dimitriadis, Drakesmith, et al. 2017b; Messaritaki et al. 2019; Clarke et al. 2020). The individual IWSBN^TF^ is a weighted brain network reflected as a symmetric 2D matrix of size 90 × 90.

#### Semi-metric analysis of the SBN

Here, we followed a network analysis over individual integrated SBN under the notion of shortest path length. Before estimating the shortest path length, we estimated the distance matrix as a transformation estimated over SBN by the following formula:

(1) \begin{equation*} {d}_{ij}=\frac{1}{{\mathrm{SBN}}_{ij}}-1, \end{equation*}

where SBN*_ij_* is the structural connectivity and *d_ij_* is the distance between nodes *i* and *j*.

In a weighted SBN, higher values are more naturally interpreted as shorter distances and the entries of SBN should consequently be inverted such that 2 brain areas with high structural connectivity are closer in the distance matrix.

SBN*_ij_* and also the Distance graph *d* do not generally yield a metric topology which means that the triangle inequality may be violated: ${d}_{ij}>{d}_{ik}+{d}_{kj}$ for some node, *k*. This means that the shortest path between 2 brain areas may not be the direct edge, but an indirect path via a number of edges. An edge is semi-metric if it connects 2 nodes via a path that includes intermediate nodes, while a metric edge connects 2 nodes directly (Galvin and Shore 1991). Figure 1.2 exemplifies the notion of semi-metric analysis for a simple brain network.

To measure the degree of semi-metric behavior in a brain network, we adopted the semi-metric ratio (SMR; Rocha 2002)

(2) \begin{equation*} {\mathrm{SMR}}_{ij}=\frac{d_{ij}}{d_{ij}^t}, \end{equation*}

where *d_ij_* is estimated by the distance matrix *d* (Fig. 2B), while ${d}_{ij}^t$ is the total distance between *i* and *j* computed over the distance matrix *d_ij_* as shortest path and via Johnson's algorithm (Fig. 2C) (Johnson 1954). ${\mathrm{SMR}}_{ij}$ is >1 for semi-metric edges and equal to 1 for metric edges (Fig. 2D). SMR is a matrix of the same size as the original integrated SBN (90 × 90), and every entry of SMR{*i*,*j*} tabulates the semi-metric behavior of every pair of brain areas (Fig. 2D). We estimated SMR in 6 spatial scales: (i) at the finest edge scale, (ii) global, (iii) left hemisphere, (iv) right hemisphere, (v) interhemispheric links, and (vi) BAI as the ratio of left and right hemispheric value by combining (iii) and (iv) (Fig. 2E). Global SMR is estimated as the percentage of semi-metric edges across the whole brain network. Left/right hemispheric SMRs are computed as the percentage of semi-metric edges within each hemisphere. Interhemispheric SMR quantifies the percentage of semi-metric edges that connect only brain regions located in different hemispheres (Fig. 2E). We estimated the BAI based on SMR as the ratio of SMR values integrated in the left hemisphere versus SMR values integrated in the right hemisphere.

(3) \begin{equation*} {\mathrm{SMR}}^{\mathrm{LH}\ \mathrm{or}\ \mathrm{RH}}=\frac{\sum_{i,j}\left({\mathrm{SMR}}_{ij}>1\right)}{\left[E\right]}, \end{equation*}

where {*i*,*j*} refer to nodes that are located within either the left or right hemisphere, a total of 45 ROIs/nodes per hemisphere.

Brain asymmetric index based on SMR index is defined as

(4) \begin{equation*} \mathrm{BAI}=\frac{{\mathrm{SMR}}^{\mathrm{LH}}}{{\mathrm{SMR}}^{\mathrm{RH}}}. \end{equation*}

A BAI > 1 reflects a leftward asymmetry while a BAI < 1 denotes a rightward asymmetry.

We also defined the semi-metric percentage (SMP), which measures the overall level of semi-metric behavior of a network or a node (brain area) as the average percentage of semi-metric paths over the total number of connections (Fig. 2F).

This measure is obtained from the SMR in the network level

(5) \begin{equation*} \mathrm{SMP}=\frac{\sum_{i,j}\left({\mathrm{SMR}}_{ij}>1\right)}{\left[E\right]}, \end{equation*}

where |*E*| is the total number of connections in the network under consideration. SMP is a vector of size equal to the number of nodes, in our case 90. SMP integrates the edge-base information of the SMR index into the ROI/node level.

We calculated the SMP index for different spatial scales as a measure of dispersed information flow between regions: (i) at the whole-brain connectome (global level; mean across 90 nodes), (ii) at the level of one hemisphere ((ii) left/(iii) right) by getting the mean SMP over 45 nodes, and (iv) node levels (Fig. 2G).

#### Group differences in sparsity, global, hemispheric, and interhemispheric strength of ISBN

In order to strengthen any potential findings under semi-metric analysis, we quantified the sparsity (number of edges divided by the total number of possible edges, here 4005 = 90 × 89/2), the global, the hemispheric, and interhemispheric strength of ISBN in both groups.

### A volume-based BAI—BAI^Vol^

We explored cortical and subcortical brain volumes based on segmentations of 90 (45 left/right) cortical gray matter and subcortical WM regions based on the AAL atlas including intracranial volume. As in our first study (Lancaster et al. 2019), segmented subcortical and cortical brain areas were visually inspected and further statistically evaluated following ENIGMA protocols (http://enigma.ini.usc.edu/protocols/imaging-protocols). Our analysis focused on two subnetworks: the language network and a subcortical network. The language network consists of the following brain areas: (i) Frontal Inferior Opercularis, (ii) Frontal Inferior pars Triangularis, (iii) Frontal Inferior Orbital, (iv) Rolandic Operculum, (v) Temporal Superior, (vi) Temporal Pole Superior, (vii) Temporal Middle, (viii) Temporal Pole Middle, (ix) Postcentral, (x) Parietal Superior, (xi) Parietal Inferior, (xii) Supramarginal, and (xiii) Angular Gyrus. We divided every brain volume by hemispheric intracranial volume, and we estimated the volumetric BAIs by first summing the hemispheric brain volumes for both subnetworks. Then, we divided the total hemispheric brain volumes between left and right hemispheres. We also estimated a BAI using the whole set of brain volumes in both hemispheres. The following equation describes the BAI^Vol^ for both subnetworks and whole brain network analysis:

(6) \begin{equation*} {\mathrm{BAI}}^{\mathrm{Vol}}=\frac{\mathrm{Lef}{\mathrm{t}}_{\mathrm{Hemispheric}}\mathrm{Brain}\ \mathrm{Volume}}{\ {\mathrm{Right}}_{\mathrm{Hemispheric}}\ \mathrm{Brain}\ \mathrm{Volume}}\ . \end{equation*}

### Statistical analysis

We adopted the Wilcoxon Rank Sum Test to compare SMR and SMP indices at every spatial scale between the low and high SCZ-PRS groups for detecting group differences. Below, we summarized in detailed the whole set of statistical analysis independently per index and spatial scale. We addressed the multiple comparison problem per index and for specific spatial scales by adopting Benjamini–Hochberg correction of False Discovery Rate (FDR; Benjamini and Hochberg 1995).

#### Statistical analysis SMR

##### Group comparison at the finest edge scale

**Fig. 3:**
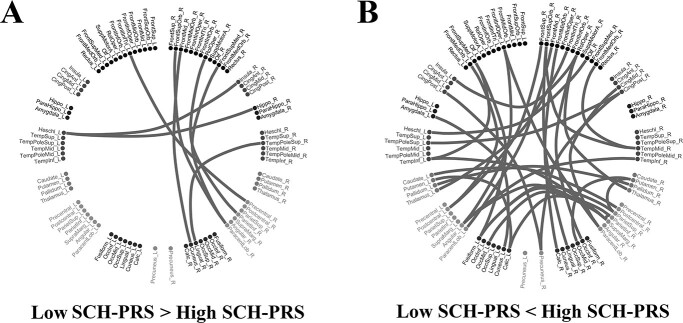
Network Topologies of Group differences in Semi-Metricity measured with SMR index at the finest edge scale. Post hoc statistical analysis of SMR index. SMR group differences are presented employing a circular distribution of the 90 ROIs of the adopted AAL template with 45 ROIs on the left semi-circle and the homologous 45 ROIs on the right semi-circle. We group ROIs into seven groups per hemisphere and the precuneus was isolated since it is a target ROI for many studies in various brain disorders/diseases A) Network topology of the higher SMR values for the low SCZ-PRS group compared to the high SCZ-PRS group. B) Network topology of the higher SMR values for the high SCZ-PRS group compared to the low SCZ-PRS group. (*P* < 0.05, corrected with FDR at the alpha = 0.05).

We compared groups of individual SMR matrices at every possible pair of brain areas 90*89/2 = 4.005 possible combinations. We addressed the multiple comparison problem by adopting Benjamini–Hochberg correction of FDR (Fig. 3).

##### Group comparisons across the (i) global, (ii) left hemisphere, (iii) right hemisphere, (iv) interhemispheric links, and (v) BAI

**Fig. 4:**
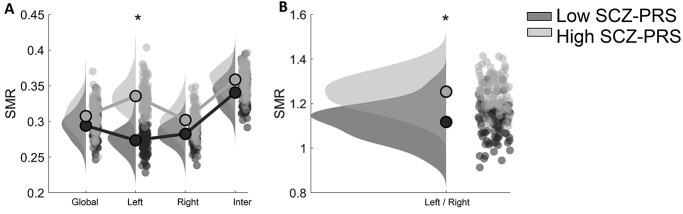
Group differences in semi-metricity measured with SMR index at various spatial scales. Post hoc statistical analysis of SMR index. (* denotes group differences with *P* < 0.05, corrected with FDR at the alpha = 0.05) A) Global, left, right, and interhemispheric scale B) Left/right hemispheric asymmetry scale.

We performed a group comparison per spatial scale (5 in total) and then we corrected for multiple comparison using FDR (Fig. 4).

#### Statistical analysis SMP

##### Group comparisons across the (i) whole-brain connectome (global level; mean across 90 nodes), and at the level of one hemisphere ((ii) left/(iii) right) by getting the mean SMP over 45 nodes

**Fig. 5:**
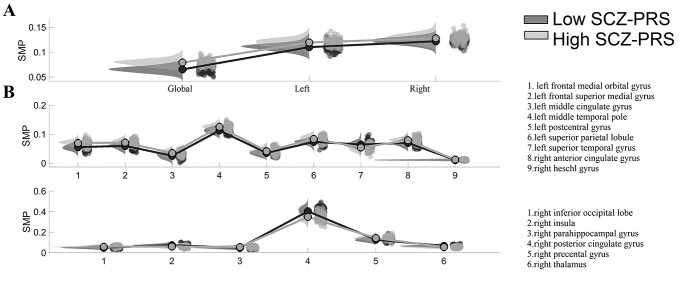
Group differences in semi-metricity measured with SMP index at various spatial scales. Post hoc statistical analysis of SMP index. A) Group comparisons in SMP in the whole-brain, left and right. No group differences were detected at that level (*P* < 0.05, corrected with FDR at the alpha = 0.05). B) Main effect of diagnosis at the nodal—brain area level. *P* < 0.05, corrected with FDR at the alpha = 0.05).

We performed a group comparison per spatial scale (3 in total) and then we corrected for multiple comparison using FDR (Fig. 5A).

##### Group comparison at the finest node scale

We compared groups of individual SMP vectors of size [1 × 90] across the node dimension and then we corrected for multiple comparison using FDR (Fig. 5B).

#### Associations between BAI and psychotic experiences

In alignment to our previous study (Lancaster et al. 2019), we estimated the associations between BAI and psychotic experiences using Firth’s Bias-Reduced Logistic Regression via the logistf package in R. This approach computes confidence intervals computed by penalized profile likelihood to control for rare events. For WISC-III, Verbal, Performance, and Total IQ from the WISC were regressed against BAI in a series of linear models. Sex was added into each model as a regressor in all cases.

#### Group by sex interaction on semi-metric topology

The group × sex interaction effect on BAI was tested via a 2-way analysis of variance (ANOVA) with sex as between factor, BAI as within-subjects factor, and age as covariate.

#### Group differences in sparsity, global, hemispheric, and interhemispheric strength of ISBN

We adopted a Wilcoxon Rank Sum Test to quantify group differences in sparsity, global, hemispheric, and interhemispheric strengths (*P* < 0.05).

#### Group differences in terms of BAI^Vol^

We adopted a Wilcoxon Rank Sum Test to quantify group differences of BAI^Vol^ in 3 different networks, and each of the targeted brain regions (*P* < 0.05).

### Code availability

Code for semi-metric analysis will become available from the following github website: https://github.com/stdimitr/semimetric_analysis_brain_networks.

### Preparation of plots

**Figure 6:**
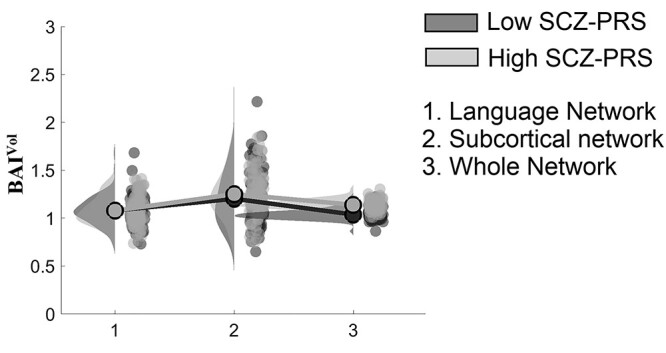
Group differences in BAI^Vol^ at various spatial scales. Post hoc statistical analysis of BAI^Vol^ index.

We adopted an open software for the visualization of the distribution of SMR/SMP values across spatial scales (Figs. 4–6) (Allen et al. 2021).

## Results

### Topology of semi-metricity measured with SMR index associated to SCZ-PRS

Figure 3A illustrates the structural connections where SMR was higher for the low SCZ-PRS group compared with the high SCZ-PRS group, while Fig. 3B demonstrates the structural connections where SMR was higher in the high SCZ-PRS group compared with the low SCZ-PRS group. The network constructed with the higher SMR values for the high SCZ-PRS group (Fig. 3B) was denser compared with the one of the low SCZ-PRS group (Fig. 3A) revealing the less constrained structural connectivity of the high SCZ-PRS group.

Figure 4 illustrates the integration of SMR into the various spatial network scales where group difference has been detected (*P* < 0.05, corrected with FDR at the alpha = 0.05). Following a detailed analysis based on the SMR index at different spatial scales, we found a higher amount of indirect connectivity of brain areas in the left hemisphere in the high SCZ-PRS group compared with the low SCZ-PRS group (Fig. 4). We observed a higher vulnerability of the left hemisphere to structural changes for the high SCZ-PRS compared with the low SCZ-PRS as measured by the BAI (Left/Right).

SMR values were integrated into global network level, in left and right hemisphere, and in interhemispheric edges. We also estimated a BAI based on SMR values as the ratio of SMR values integrated into the left hemisphere versus SMR values integrated into the right hemisphere. No group differences were detected at the global network level, in the right hemisphere, and in the interhemispheric connections, while group differences with higher values for the SCZ-PRS group were revealed in the left hemisphere, and in the ratio left versus right hemisphere which served as a BAI (*P* < 0.05, corrected with FDR at the alpha = 0.05).

### Semi-metricity measured with the SMP index at different spatial scales driven from SCZ-PRS

No group differences were revealed at the 3 spatial scales based on SMP estimations (Fig. 5A). In contrast, group comparisons of the nodal SMP estimates yielded increased SMP values in 11 brain areas for the high SCZ-PRS group compared with the low SCZ-PRS group and in 4 brain areas for the reverse contrast (Fig. 5B). Those 15 brain regions are shown topologically in Supplementary Fig. 1.

### Group differences in sparsity, global, hemispheric, and interhemispheric strength of ISBN

Our analysis revealed no group differences of ISBN in terms of sparsity, global, hemispheric, and interhemispheric strength (Table 2).

**Table 2:** Group differences in terms of sparsity, global, hemispheric, and interhemispheric strength of ISBN.

	Low SCZ-PRS	High SCZ-PRS	*P*
Sparsity	0.13 ± 0.005	0.13 ± 0.005	0.98
Global Strength	447.03 ± 15.89	449.19 ± 17.41	0.15
Left hemispheric strength	97.90 ± 21.32	97.45 ± 18.77	0.84
Right hemispheric strength	102.12 ± 16.78	104.01 ± 17.26	0.51
Interhemispheric strength	123.49 ± 15.43	123.86 ± 15.65	0.88

### Psychopathology, cognition, and brain asymmetry

For individuals where psychotic experiences data were available (*n* = 172), we observed no nominal association between an increased incidence of psychotic experiences and high brain asymmetry group allocation (*P* = 0.535). For individuals where SCZ-PRS and WISC-III measures were available (*n* = 183), we observed no association between SCZ-RPS and any IQ dimension (Table 3).

**Table 3:** OR and β coefficients (±95% confidence intervals) for psychotic experiences and WISC-III IQ measures by BAI (higher OR/coefficients reflect an association with the high SCZ-PRS group).

Phenotype	Estimate	Lower 0.95%	Upper 0.95%	*P*
Psychotic experiences	1.061^a^	1.0014	1.1251	0.535
WISC-III (verbal)	0.911^b^	−4.4016	6.2216	0.737
WISC-III (performance)	−3.978^b^	−9.5560	1.6160	0.165
WISC-III (total)	−2.036^b^	−7.4984	3.4384	0.467

^a^Odds ratio (OR).

^b^β coefficients.

### Group by sex interaction on semi-metric topology

The group × sex interaction effect on BAI was tested via a 2-way analysis of variance (ANOVA) with sex as between factor, BAI as within-subjects factor, and age as covariate. Our findings revealed no group × sex interaction effects on BAI (*P* = 0.527; *F*-statistic = 0.56).

### Group differences in BAI^Vol^

Our statistical analysis showed group differences in BAI^Vol^ in one out of three networks (Fig. 6: language network: *P* = 0.11, subcortical network: *P* = 0.81, whole-network: *P* = 9.54 × 10^−25^).

## Discussion

This study examined indirect (semi-metric) structural connectivity via dMRI in young adults from a population cohort with either low or high burden of common risk alleles for SCZ. SMRs were calculated to assess the proportion of indirect shortest functional pathways at global, hemisphere, network, and edge level, and SMPs at global, hemisphere, network, and node levels. We defined a total of 6 SMR-based indices and 4 SMP-based indices, based on different spatial scales. We estimated SMR in 6 spatial scales: (i) at the finest edge scale, (ii) global, (iii) left hemisphere, (iv) right hemisphere, (v) interhemispheric links, and (vi) BAI as the ratio of left and right hemispheric value by combining (iii) and (iv).The SMR analysis on the fine edge scale revealed a denser network with higher SMR values for the high SCZ-PRS > low SCZ-PRS. A detailed analysis of SMR values at different spatial scales revealed a higher proportion of indirect communication of brain areas in the left hemisphere in the high SCZ-PRS group compared with the low SCZ-PRS group. We observed no group differences within the right-hemisphere and the interhemispheric connections. Finally, our findings indicate a higher vulnerability of the left hemisphere to structural changes for the high SCZ-PRS compared with the low SCZ-PRS as measured by the BAI.

Our results could not be explained by any group difference in terms of sparsity, global, hemispheric, and interhemispheric strength estimated over individual ISBNs. Moreover, we observed no nominal association between an increased incidence of psychotic experiences and high brain asymmetry group allocation, and no association between SCZ-PRS and any IQ dimension (Table 3). Our findings furthermore revealed no group × sex interaction effects on BAI. Interestingly, BAI^Vol^ in 2 subnetworks and in the whole network revealed no group differences in language and subcortical targeted network but showed a higher leftward volumetric brain asymmetry for the high SCZ-PRS group compared with the low SCZ-PRS group.

Left–right brain asymmetry is an important characteristic of normal human brain organization supporting specific cognitive and sensory somatotopic functions such as left-hemisphere dominance for language and right-handedness (Guadalupe et al. 2017; Kong et al. 2018). Above 90% of individuals show these dominances (Toga and Thompson 2003; Karolis et al. 2019). A possible role of altered structural and functional brain asymmetry in schizophrenia has been studied for several decades (Petty 1999; Berlim et al. 2003; Oertel et al. 2010; Ribolsi et al. 2014). Theoretical work investigated the relationship of disrupted laterality for language to disorganized speech and auditory hallucinations (Sommer et al. 2001; Ocklenburg et al. 2015). Individuals with SCZ showed a decreased left-lateralized language dominance (Spironelli et al. 2008), while the rate of non-right-handedness in schizophrenia is elevated compared with the general population (Hirnstein and Hugdahl 2014). Interestingly, some genomic loci that affect parts of structural brain asymmetry or hand predominance overlap with those associated with SCZ (Wiberg et al. 2019; Cuellar-Partida et al. 2021; Sha, Schijven, Carrion-Castillo, et al. 2021a; Sha, Schijven, Francks 2021b). A recent large-scale MRI study from the ENIGMA consortium revealed lifespan trajectories of asymmetrical brain development that may potentially contribute to the pathophysiology of SCZ (Schijven et al. 2022).

Our findings might indicate a less efficient structural connectivity in the higher genetic risk group. This is the first study in a population-based sample that reveals differences in the efficiency of SBNs associated with common genetic risk variants for SCZ. To establish a clinical importance of our findings, we have to repeat the whole analysis in a sample of similar age and gender distribution as the one analyzed here. Afterward, we can evaluate, if present, findings that show little or no genetic overlap with SCZ common variant liability as with subcortical structural abnormalities (Ranlund et al. 2018; Lancaster et al. 2019; Lv et al. 2021). In an SCZ cohort, if we detect associations between SCZ-PRS and individual deviations from the normative range of BAI from the healthy control group of low SCZ-PRS, then, we can argue that present findings are of high clinical importance (Lv et al. 2021).

Semi-metric analysis of the functional connectome is sensitive and specific to psychopathologies (Peeters et al. 2015; Simas et al. 2015; Simas and Rocha 2015; Suckling et al. 2015). Simas and Rocha (2015) untangled both positive and negative deviations in the network level proportion of semi-metric connections in autism and major depressive disorder respectively compared with neurotypical healthy controls (Simas et al. 2015). Peeters et al. (2015) revealed that psychosis was associated with only positive changes to semi-metricity and that the severity of symptoms related to the magnitude of change (Peeters et al. 2015). In Alzheimer's disease (AD), both positive and negative effects were observed, where semi-metric analysis showed that AD subjects have higher topological semi-metric intersubject variability compared with healthy controls (Suckling et al. 2015). The characterization of AD as a disconnection syndrome does not reflect the isolation of one or more brain areas but is expressed topologically with an increment of the global indirect functional pathways (Suckling et al. 2015). Guo et al. (2019) analyzed resting-state fMRI Pearson’s correlation networks from both a healthy control group and a group of subjects with autism. They found an increment of indirect functional connections in the adult autistic brain (Guo et al. 2019).

These above studies show that in healthy individuals, functional MRI (fMRI) networks derived from Pearson’s correlation metric show semi-metric behavior for up to 80% of their connections (Simas et al. 2015). However, due to a limited number of studies followed a semi-metric analysis, it is difficult to define the normal range of the percentage of semi-metric connections in both local and global level and also across the lifespan. There is strong evidence that the degree of semi-metricity (i.e. like transitivity) in anatomical/structural networks predicts functional connectivity (Goñi et al. 2014). The aforementioned studies suggest that an optimum value of semi-metricity exists both globally and locally that is also linked to healthy brain function. In addition, different disorders have different patterns of change (positive or negative or both effects) relative to control samples. Based on these studies, we can assume that there is an optimum level of semi-metricity at every spatial network scale associated with healthy brain function.

Our data thus point to a higher degree of indirect connectivity particularly within the left hemisphere in the high genetic risk group. Such indirect connectivity could indicate vulnerability to less efficient structural and functional integration via alternative pathways. Our findings are in line with structural imaging studies focusing on WM alterations in SCZ (Miyata et al. 2012; Ribolsi et al. 2014). In general, research findings in SCZ patients based on DTI have supported the hypothesis of a more vulnerable left hemisphere compared with the right hemisphere in SCZ (Oertel-Knöchel and Linden 2011), which is positively correlated to an increase in positive symptoms of psychosis (Fujiwara et al. 2007; Kunimatsu et al. 2008; Miyata et al. 2012; Ribolsi et al. 2014).

Our findings are also compatible with the general pattern of volume loss documented in structural imaging studies in schizophrenia. A meta-analysis concluded that the pathological processes underlying cortical gray matter loss in SCZ was more active in the left than in the right hemisphere, which could explain the abnormal asymmetries seen in these patients (Vita et al. 2012). Further analysis is needed at the microstructural level to further explain our findings. A recent study suggested that global gray matter asymmetry is related to the concept of developmental stability, which refers to the capacity of an organism to buffer its development against genetic or environmental perturbations (Núñez et al. 2017). These researchers reported that global gray matter asymmetry is a useful indicator of perturbations during neurodevelopment, and we would suggest that the same is true for metrics of WM connectivity, as analyzed in the present study, as well.

In summary, high SCZ-PRS was associated with increased indirect connections between brain areas in the left hemisphere based on longer shortest structural pathways identified by graph analysis. Such changes in connectivity could drive alterations in hemispheric functional asymmetry implicated in the pathophysiology of SCZ. These are the first results suggesting altered structural connectivity in healthy individuals stratified by the SCZ-PRS. Replication of these findings in a follow-up study would thus be important.

## Conclusion and implications

This is the first study in a general population-based sample that suggests a potential reduction of the efficiency of left-hemispheric communication associated with increased genetic risk for SCZ. It thus contributes to the understanding of the mechanisms by which (poly-)genetic variation confers vulnerability to SCZ. To further our mechanistic understanding, the adopted semi-metric analysis should also be applied to functional MRI data in order to study the association between the efficiency of structural and functional connectivity.

## Author Contributions

DL, KS, MOD, MJO, PH: general study design.

GP, SID. worked on data acquisition.

The conception of the research analysis: SID;

Imaging Methods and design: SID;

Data analysis: SID;

Drafting the manuscript: SID.

Critical revision of the manuscript:

All authors;

Every author read and approved the final version of the manuscript.

## Supplementary Material

Supp_material_bhac256
