# Supplementary material for: Genetic risk for schizophrenia is associated with increased proportion of indirect connections in brain networks revealed by a semi-metric analysis: evidence from population sample stratified for polygenic risk
Source: Cereb Cortex. 2022 Jul 14;33(6):2997–3011. doi: 10.1093/cercor/bhac256 (PMC10016061; doi:10.1093/cercor/bhac256)
Supplement: Supp_material_bhac256 [file supp_material_bhac256.docx]

**SUPPLEMENTARY MATERIAL**

**Title:**

Genetic Risk for Schizophrenia is associated with Increased Proportion of Indirect Connections in Brain Networks revealed by a semi-metric analysis: Evidence from Population Sample Stratified for Polygenic Risk

**Authors:**

Dimitriadis, S.I^1-4*^ ,Perry G^2^, Lancaster TM^1,2,7^, Tansey K.E^5^ , Singh K.D^2^, Holmans P^3^,Pocklington A^3^, Davey Smith, G.^5,6^,Zammit S ^3,6^, Hall J ^1,3^, O’Donovan, M.C ^1, 3^, Owen M.J ^1,3^, Jones D.K^2^, Linden, D.E ^1-3,6^

**Affiliations:**

^1^Neuroscience and Mental Health Research Institute, Cardiff University, Cardiff, UK.

^2^ Cardiff University Brain Research Imaging Centre (CUBRIC), School of Psychology, College of Biomedical and Life Sciences, Cardiff University, Cardiff, UK

^3^ MRC Centre for Neuropsychiatric Genetics and Genomics, Division of Psychological Medicine and Clinical Neurosciences, Cardiff School of Medicine, Cardiff University, Cardiff, UK

^4^ Neuroinformatics Group, School of Psychology, Cardiff University, Cardiff, UK

^5^ MRC Integrative Epidemiology Unit (IEU) at the University of Bristol, Bristol, UK.

^6^Population Health Sciences, Bristol Medical School, University of Bristol, Bristol, United Kingdom

^7^Department of Psychology, Bath University, Bath, United Kingdom

Correspondence:DimitriadisS@cardiff.ac.uk ; stidimitriadis@gmail.com


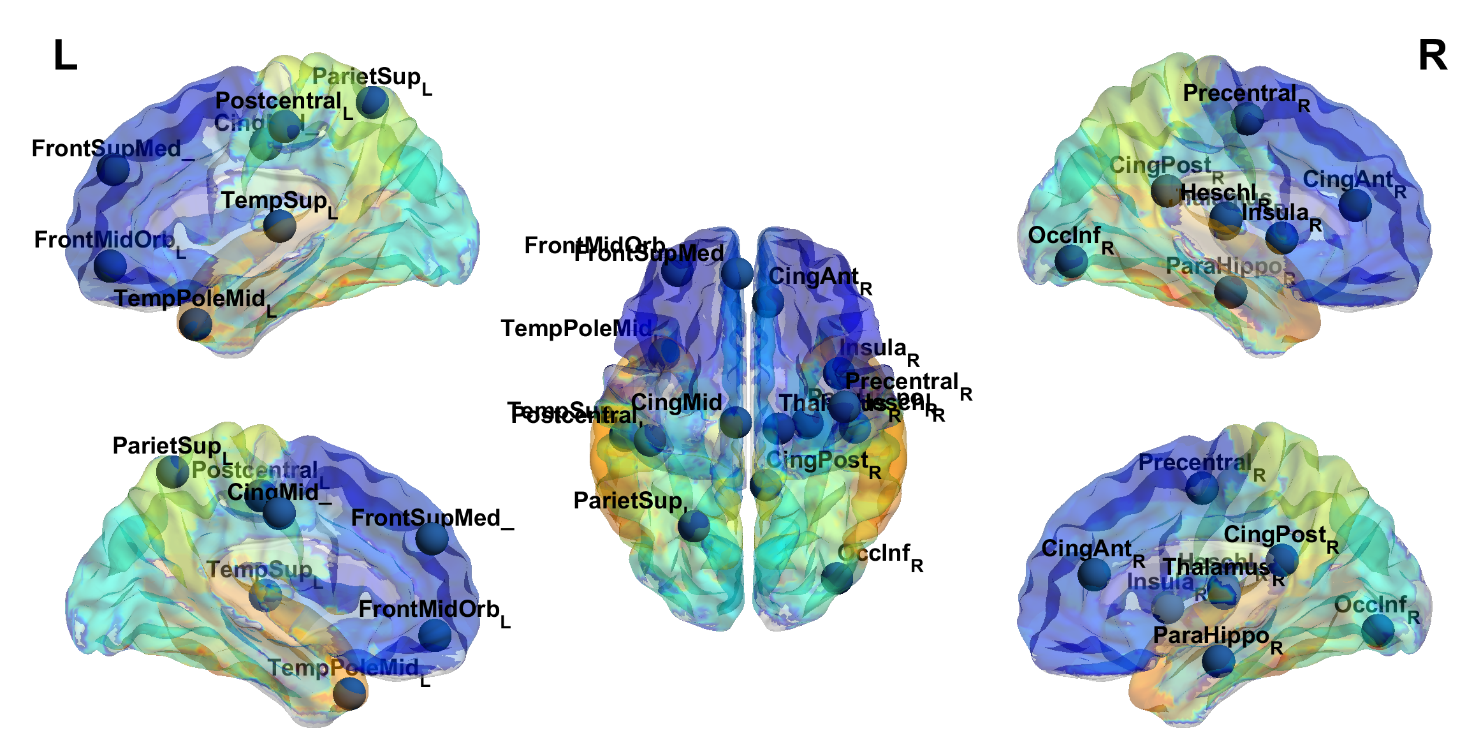


**S1**. Topological layout of the fifteen brain regions identified in nodal SMP group comparisons showed in Fig.5. The figure has been designed with BrainNetViewer software (Xia et al., 2013).

**Reference**

Xia M, Wang J, He Y (2013) BrainNet Viewer: A Network Visualization Tool for Human Brain Connectomics. PLoS ONE 8(7): e68910. https://doi.org/10.1371/journal.pone.0068910
